# A theoretical derivation of response to selection with and without controlled mating in honeybees

**DOI:** 10.1186/s12711-021-00606-5

**Published:** 2021-02-17

**Authors:** Manuel Du, Richard Bernstein, Andreas Hoppe, Kaspar Bienefeld

**Affiliations:** Institute for Bee Research Hohen Neuendorf, Friedrich-Engels-Str. 32, 16540 Hohen Neuendorf, Germany

## Abstract

**Background:**

In recent years, the breeding of honeybees has gained significant scientific interest, and numerous theoretical and practical improvements have been made regarding the collection and processing of their performance data. It is now known that the selection of high-quality drone material is crucial for mid to long-term breeding success. However, there has been no conclusive mathematical theory to explain these findings.

**Methods:**

We derived mathematical formulas to describe the response to selection of a breeding population and an unselected passive population of honeybees that benefits indirectly from genetic improvement in the breeding population via migration. This was done under the assumption of either controlled or uncontrolled mating of queens in the breeding population.

**Results:**

Our model equations confirm what has been observed in simulation studies. In particular, we have proven that the breeding population and the passive population will show parallel genetic gain after some years and we were able to assess the responses to selection for different breeding strategies. Thus, we confirmed the crucial importance of controlled mating for successful honeybee breeding. When compared with data from simulation studies, the derived formulas showed high coefficients of determination $> 0.95$ in cases where many passive queens had dams from the breeding population. For self-sufficient passive populations, the coefficients of determination were lower ($\sim 0.8$) if the breeding population was under controlled mating. This can be explained by the limited simulated time-frame and lower convergence rates.

**Conclusion:**

The presented theoretical derivations allow extrapolation of honeybee-specific simulation results for breeding programs to a wide range of population parameters. Furthermore, they provide general insights into the genetic dynamics of interdependent populations, not only for honeybees but also in a broader context.

## Background

The global ecological and economic importance of honeybees, mainly due to their pollination activities, has long been recognized [1–4]. In spite of their importance, systematic breeding of honeybees for economically important traits, such as honey yield or gentleness, which are based on standardized performance tests, is only a very recent activity in Europe and currently limited to only a few areas [5, 6]. Establishing new animal breeding programs always comes with a need for various aspects of breeding infrastructure [7]. A particular difficulty in honeybee breeding is to provide a controlled mating process. This is due to the reproductive peculiarities of this species, including multiple mating in the air which usually cannot be observed, let alone controlled [6, 8]. Controlled mating can be achieved by artificial insemination or by the use of isolated mating stations, where geographic seclusion allows the mating drones to be restricted to the descendants of few drone producing colonies. Typically, the queens of these drone producing colonies share a common dam.

Both these possibilities come with high maintenance costs [6, 9]. Consequently, there have been many attempts to breed honeybees by selecting only in the (maternal) queen path, while neglecting mating control and thus the influence of the (paternal) drones [10–13]. However, recent simulation studies have shown that controlled mating is a crucial component of successful honeybee selection schemes [14]. In particular, these studies showed that breeding without paternal selection is at most half as effective as breeding with controlled mating. Especially, when a small breeding population was faced with a large unselected passive population, the limitations were enormous. In the extreme case, when the relative proportion of the breeding population to the whole population tended to zero, no genetic gain whatsoever could be observed after a few generations. Without controlled mating, the situation could be improved only slightly by distributing genetic material from the breeding population to the unselected surrounding population. Finally, it was shown that through exchange of genetic material, the passive population could benefit greatly from improved breeding schemes in the breeding population.

To our knowledge, the simulation studies in [14] were the first attempt to theoretically model solely maternal selection strategies in honeybees. In particular, to date, there is no comprehensive analytical model to explain their outcomes. Such a model would allow the simulation results of [14], which were obtained for a limited set of population sizes and genetic exchange rates, to be extrapolated to a wide range of actual honeybee populations. Thus, the practical applicability of the knowledge gained from the simulations would be greatly enhanced.

Before the era of computer simulations, analytical derivations were the only possibility to predict the limits of selection [15], losses of genetic variance [16], or expected genetic response [17] in breeding schemes. The vastly increasing power of computers has since led to a plethora of simulation studies in livestock breeding, each yielding answers to a few very specific questions. However, while simulation studies are a strong tool to predict *how* specific systems, such as breeding populations, behave under predefined circumstances, they have only limited power in explaining the *why* behind a certain behavior. Therefore, mathematical explanations for empirical observations remain important to this day, because they allow the plausibility of results to be examined and provide further insight into the mechanisms behind the data, thus allowing for generalizations beyond the scope of concrete simulation studies.

In honeybee breeding, the population may be divided into an active breeding population and an unselected passive population, which resembles the structure of nucleus breeding schemes in other livestock species, where only an elite nucleus group of animals undergoes selection and provides a larger commercial group with genetic material. In the late 1950s, Smith [18–20] showed under simplifying assumptions that both sub-populations would eventually show similar rates of genetic gain, with the commercial stock lagging behind in time. These studies were later formalized and extended by Bichard [21]. Building on Bichard’s work, James [22] developed a mathematical model for the genetic progress in the nucleus and general population in breeding schemes with genetic exchange in both directions.

Although all aforementioned studies assumed controlled mating, similar theory can be developed to model genetic change in related settings, such as breeding with or without controlled mating in honeybees. This needs to take the genetic attributes and mating behavior of honeybees into account. Specifically, in honeybees, the male drones develop from unfertilized eggs and are haploid, whereas the female queens and workers are diploid. After hatching, a young queen mates in mid-air with up to 20 drones from other hives and stores their sperm in her spermatheca. The sperm from that nuptial flight is henceforth used to fertilize the eggs from which the queen’s female offspring evolve. In this work, we provide a mathematical derivation of expected response to selection with and without controlled mating and show the implications for the unselected passive population. The results provide a theoretical justification of the results obtained in [14] and enhance their applicability to more general populations of honeybees.

## Methods

### Analytical approach

For our analytical approach, we mainly follow the gene-flow method [23, 24], which is adapted to the situation of honeybee breeding. The honeybee population is assumed to be subdivided in two groups: a breeding population that is undergoing some sort of selection, and a passive population that remains unselected but potentially benefits from genetic material that is introduced from the breeding population. We call the colonies (consisting of queen and workers) of the breeding population *breeding colonies* and those of the passive population *passive colonies*. Accordingly, we also speak of *breeding queens* and *passive queens*. Each colony is associated with the birth year of its queen, which is also the year in which the queen mates. While we assume that passive queens always mate uncontrolled, we distinguish two cases for the breeding queens: (a) uncontrolled mating of breeding queens and (b) controlled mating of breeding queens on isolated mating stations.

**Table 1:** Variable definitions

Variable	Definition
*t*	Subscript referring to the year. If variables are used without this subscript it is assumed that they are constant for all years
$B_t$ / $P_t$	Average true breeding value of breeding/passive colonies of year *t* $=B_t-B_{t-1}$ (resp. $=P_t-P_{t-1}$) Annual genetic gain among the breeding/passive colonies $=B_t-P_t$.
$\Delta B_t$ / $\Delta P_t$	
$D_t$	Genetic lag between the breeding population and the passive population
*T*	Time lag, time it takes for the passive population to reach the genetic level of the breeding population
$p_t$, *p*	Probability that a sire drone in an uncontrolled mating comes from a breeding colony
$q_t$, *q*	Proportion of passive queens with a dam queen from the breeding population
$S_{1,t}$, $S_1$	Genetic selection differential of colonies selected for breeding queen production
$S_{2,t}$, $S_2$	Genetic selection differential of colonies selected for DPQ production
$N_b$ / $N_p$	Number of breeding/passive colonies per year
$h^2_m$ / $h^2_d$	Maternal/direct heritability
$r_{md}$	Correlation between maternal and direct effects

The true breeding value of a colony is considered to be the breeding value of the worker group of that colony and is equal to the expected breeding value of a queen reared from that colony [25, 26]. For each year *t*, we denote the average true breeding values of breeding colonies and passive colonies of that year by $B_t$ and $P_t$, respectively. (See Table 1 for an overview of all the variables used). Among the group of breeding colonies in year *t*, some will be selected for reproduction. This may happen for two purposes. First, breeding colonies may be selected to produce the next generation of breeding queens (purpose 1) and, secondly, in the case of controlled mating, they may be selected to produce the sister group of drone producing queens (DPQ) on a mating station (purpose 2). Since colonies are typically selected for superior breeding values, it is reasonable to assume that the average true breeding value of the selected breeding colonies in a year is higher than the average breeding value of breeding colonies in that year. We denote the average true breeding values of the colonies of year *t* that are selected for the two purposes by $B_t+S_{1,t}$ and $B_t+S_{2,t}$, respectively. Parameters $S_{1,t}$ and $S_{2,t}$ are comparable to maternal and paternal genetic selection differentials in other livestock species.

**Fig. 1:**
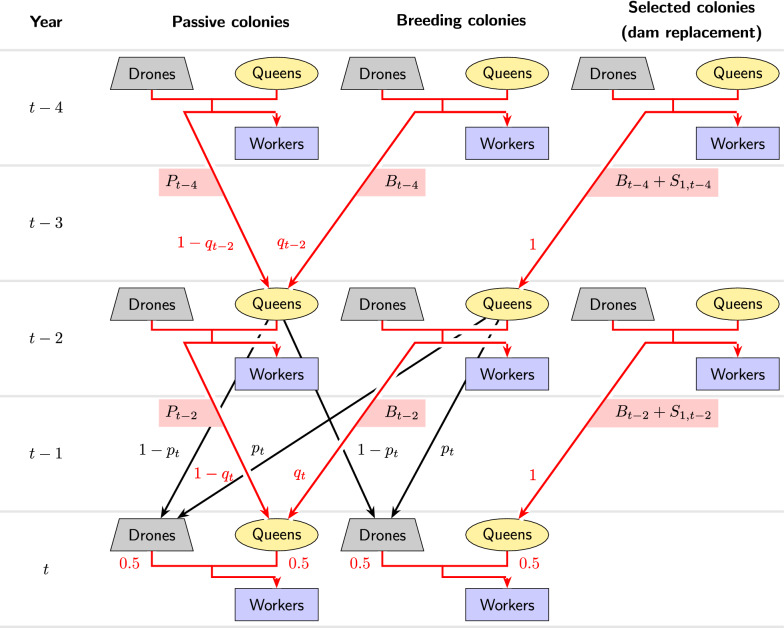
Illustration of the genetic contributions of the breeding and passive colonies with uncontrolled mating. This figure motivates the recursion formulas Eq. 1 and 3. Worker groups in year *t* receive their breeding values in equal parts from their queens and the drones that are mating partners of the queens. Passive queens in year *t* receive their breeding values either from unselected breeding colonies of year $t-2$ (probability $q_t$) or from passive colonies of year $t-2$ (probability $1-q_t$). In the former case, the average inherited breeding value is $B_{t-2}$; in the latter case, it is $P_{t-2}$. Breeding queens inherit their breeding values from selected breeding colonies of year $t-2$, with average breeding values equal to $B_{t-2}+S_{1,t-2}$. Drones which mate with breeding or passive queens in year *t* may be offspring (and thus carry the breeding values) of either unselected breeding queens (probability $p_t$) or passive queens (probability $1-p_t$) of year $t-2$. For the breeding values of unselected breeding and passive queens of year $t-2$, the same considerations apply as for those of year *t*

With uncontrolled mating, a newborn queen is assumed to be two years younger than her dam and to mate with drones from two-year-old queens (Fig. 1). When a queen in year *t* mates uncontrolled, we denote the probability for an involved drone to come from a breeding colony by $p_t$; consequently the probability for the drone to come from a passive colony is $1-p_t$. In particular, we assume that the probability $p_t$ for a queen in a free mating condition to mate with a breeding drone is independent of the sub-population of the queen. To model the dissemination of offspring from breeding queens to the passive population, we assume that a relative number $q_t$ of passive queens of year *t* have a breeding queen as their dam. Dams of breeding queens are always queens from selected breeding colonies.

**Fig. 2:**
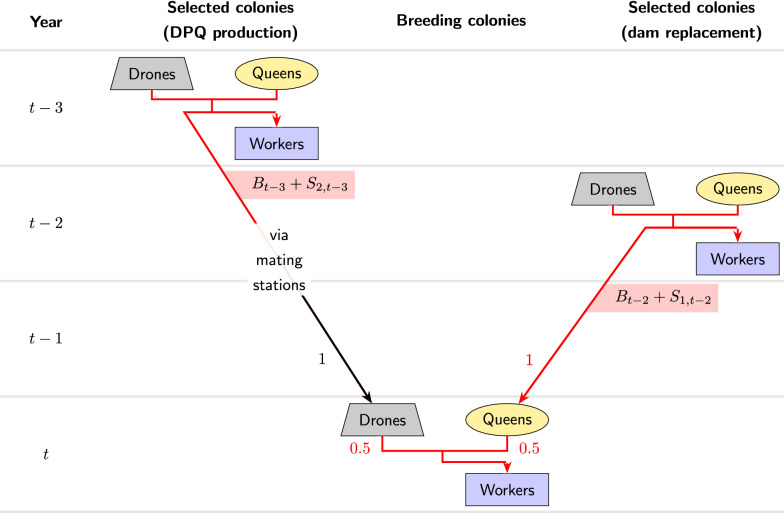
Illustration of the genetic contributions to the breeding colonies with controlled mating. This figure motivates the recursion formula Eq. 5. Breeding colonies in year *t* receive half of their genetic material from their queens which as in the case of uncontrolled mating have an average breeding value equal to $B_{t-2}+S_{1,t-2}$. The other half is inherited from the drones on a mating station whose common granddam on average has a breeding value equal to $B_{t-3}+S_{2,t-3}$

**Fig. 3:**
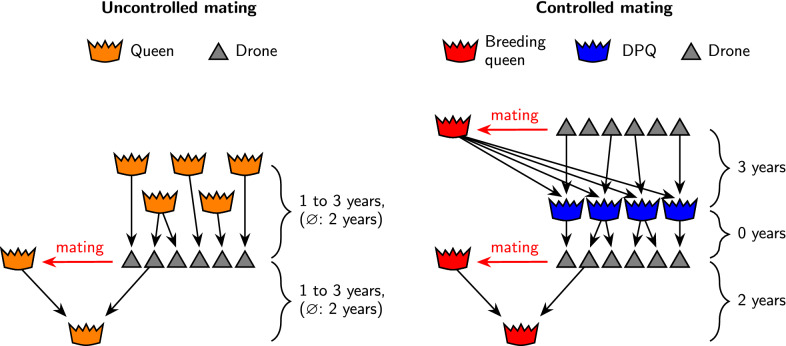
Illustration of the generation intervals in the simulations with uncontrolled or controlled mating of queens

If mating in the breeding population is controlled (Fig. 2), we still assume a generation interval of two years between queens and their dams. For the paternal inheritance path, we assume that a queen which mates controlled on a mating station is three years younger than the dam of the drone producing queens on that mating station. Controlled mating in the breeding population does not affect the paths of inheritance for the passive population described above and in Fig. 1.

In the following, we investigate the development of the variables $B_t$ and $P_t$ over time. Particularly, we are interested in the annual genetic progress rates $\Delta B_t=B_t-B_{t-1}$ and $\Delta P_t=P_t-P_{t-1}$, as well as the genetic lag $D_t=B_t-P_t$ between the breeding population and the passive population.

#### Recursive equations with uncontrolled mating

First, we consider breeding colonies with uncontrolled mating (Fig. 1). A worker group *W* in year *t* receives half of its genetic material from its queen *Q*, which in turn is an offspring from a selected colony in year $t-2$, and thus on average, has a breeding value equal to $B_{t-2}+S_{1,t-2}$. The other half of its genetic material comes from the drones which *Q* mated with. The drone producing queens are unselected and from year $t-2$. With probability $p_t$, they are breeding queens of that year and thus have an average breeding value equal to $B_{t-4}+S_{1,t-4}$. With probability $1-p_t$, they are passive queens of year $t-2$. In this case, a further distinction is necessary, since the colony from which a passive queen originates may be an unselected breeding colony (probability $q_{t-2}$) with an average breeding value equal to $B_{t-4}$ or a passive colony (probability $1-q_{t-2}$) with an average breeding value equal to $P_{t-4}$. Combining all paths of inheritance, we arrive at the following recursive equation for the average true breeding values in the breeding population:

1 $$\begin{aligned} B_t=\,&\frac{B_{t-2}+S_{1,t-2}}{2}\nonumber \\&+\frac{p_t(B_{t-4}+S_{1,t-4})}{2}\nonumber \\&+\frac{(1-p_t)(q_{t-2}B_{t-4}+(1-{q_{t-2}})P_{t-4})}{2}. \end{aligned}$$

If we assume that $p_t=p$, $q_t=q$, and $S_{1,t}=S_1$ are constant over years, grouping terms for breeding and passive populations yields:

2 $$\begin{aligned} B_t=\,&\frac{1}{2} B_{t-2}+\frac{p+q-pq}{2} B_{t-4}\nonumber \\&+\frac{(1-p)(1-q)}{2} P_{t-4}+\frac{1+p}{2} S_1. \end{aligned}$$

In the passive population, a worker group *W* in year *t* receives half of its genetic material from its queen *Q*, which in turn comes either from an unselected breeding colony (probability $q_t$) with average true breeding value $B_{t-2}$, or from a passive colony (probability $1-q_t$) with average breeding value $P_{t-2}$ (Fig. 1). Therefore, *Q* has an average breeding value that is equal to $q_tB_{t-2}+(1-q_t)P_{t-2}$. For the paternally inherited genetic material, the same considerations as with uncontrolled mating for the breeding population apply. This yields:

3 $$\begin{aligned} P_t = \,&\frac{q_tB_{t-2}+(1-q_t)P_{t-2}}{2}\nonumber \\&+\frac{p_t(B_{t-4}+S_{1,t-4})}{2}\nonumber \\&+\frac{(1-p_t)(q_{t-2}B_{t-4}+(1-{q_{t-2}})P_{t-4})}{2}, \end{aligned}$$

which in analogy to Eq. 2, again under the assumption of constant $p_t=p$, $q_t=q$, and $S_{1,t}=S_1$, can be rearranged to

4 $$\begin{aligned} P_t = \,&\frac{q}{2} B_{t-2}+\frac{p+q-pq}{2} B_{t-4}+\frac{1-q}{2} P_{t-2}\nonumber \\&+\frac{(1-p)(1-q)}{2} P_{t-4}+\frac{p}{2} S_1. \end{aligned}$$

#### Recursive equations with controlled mating

Next, we consider controlled mating in the breeding population (Fig. 2). Again, the maternally inherited part of the true breeding value of a breeding colony of year *t* is on average $\frac{B_{t-2}+S_{1,t-2}}{2}$. However now, the paternal genetic material comes from drone producing queens, which come from a selected breeding colony of year $t-3$, which on average has a breeding value equal to $B_{t-3}+S_{2,t-3}$. As a result, assuming $S_{1,t}=S_1$ and $S_{2,t}=S_2$ to be constant:

5 $$\begin{aligned} B_t=\,\frac{1}{2} B_{t-2}+\frac{1}{2} B_{t-3}+\frac{S_1+S_2}{2}. \end{aligned}$$

Recursive computation of the average breeding value of the passive population is not affected by the type of mating in the breeding population. Thus, also in the case of controlled mating in the breeding population, the average true breeding values of the passive colonies are described by Eq. 4.

#### Solving the recursive equations with uncontrolled mating

The recursive equations given by Eq. 2 and Eq. 4 are linked and thus cannot be solved independently. Therefore, first we calculate the genetic lag $D_t=B_t-P_t$. Subtracting Eq. 4 from Eq. 2 and simplifying terms results in:

6 $$\begin{aligned} D_t&=\,\frac{1-q}{2} B_{t-2}-\frac{1-q}{2} P_{t-2}+\frac{1}{2} S_1\nonumber \\&=\frac{1-q}{2} D_{t-2}+\frac{1}{2} S_1. \end{aligned}$$

For large *t*, this recursive equation converges, and its asymptotic value can be obtained by equating $D_{t-2}=D_t$ in Eq. 6:

7 $$\begin{aligned} D_t =\,\frac{S_1}{1+q}. \end{aligned}$$

This means that the genetic lag between the breeding population and the passive population is given by the genetic selection differential $S_1$ if the passive population is self-sufficient in terms of queen replacement ($q=0$). By rearing passive queens from breeding colonies ($q > 0$), this genetic lag can be reduced by up to 50% (for $q=1$).

Next, we examine the development of the breeding population. From Eq. 7, we know that for sufficiently large *t*, we can replace $P_{t-4}$ in Eq. 2 by $B_{t-4}-\frac{S_1}{1+q}$, which results in:

8 $$\begin{aligned} B_t=\,\frac{1}{2} B_{t-2}+\frac{1}{2} B_{t-4}+\frac{p+q}{1+q} S_1. \end{aligned}$$

From Eq. 8, we derive:

9 $$\begin{aligned} \Delta B_t = \,&\frac{1}{2} B_{t-2}+\frac{1}{2} B_{t-4}+\frac{p+q}{1+q} S_1 - B_{t-1}\nonumber \\ =&-\Delta B_{t-1}-\frac{1}{2}\Delta B_{t-2}-\frac{1}{2}\Delta B_{t-3}\nonumber \\&+\frac{p+q}{1+q} S_1. \end{aligned}$$

The stable value of $\Delta B_t$ for sufficiently large values of *t* can be obtained from equating $\Delta B_t=\Delta B_{t-1}=\Delta B_{t-2}=\Delta B_{t-3}$ in Eq. 9, resulting in:

10 $$\begin{aligned} \Delta B_t =\,\frac{p+q}{3+3q} S_1. \end{aligned}$$

Since the genetic lag $D_t$ is constant for large *t*, the annual rate of genetic improvement in the passive population has to be equal to that of the breeding population:

11 $$\begin{aligned} \Delta P_t = \frac{p+q}{3+3q} S_1. \end{aligned}$$

The annual rate of genetic progress in the breeding and passive populations can thus range from 0 ($p=q=0$) to $\frac{S_1}{3}$ ($p=1$). For a fixed probability $p<1$ of queens to mate with drones from breeding colonies, the rate of genetic progress increases slightly with increasing *q*, i. e. when more passive queens originate from breeding colonies.

From the annual genetic gain $\Delta B_t=\Delta P_t$ and the genetic lag $D_t$, one can calculate the time lag between the breeding and the passive populations, i.e. how many years the genetic level of the passive population lags behind the genetic level of the breeding population. This value amounts to:

12 $$\begin{aligned} T=\frac{D_t}{\Delta B_t}=\frac{3}{p+q}. \end{aligned}$$

Thus, the time lag is at least 1.5 years but may become arbitrarily long if the probability for drones from breeding colonies to reproduce is low and few passive queens originate from breeding colonies.

#### Solving the recursive equations with controlled mating

In the case of controlled mating, the genetic progress in the breeding population does not depend on the passive population (see Eq. 5). Thus, we can calculate annual genetic progress in the breeding population directly as:

13 $$\begin{aligned} \Delta B_t&=\frac{1}{2} B_{t-2}+\frac{1}{2} B_{t-3}+\frac{S_1+S_2}{2}-B_{t-1}\nonumber \\&=-\Delta B_{t-1}-\frac{1}{2} \Delta B_{t-2}+\frac{S_1+S_2}{2}, \end{aligned}$$

and solve this recursive equation analogous to Eq. 10 as:

14 $$\begin{aligned} \Delta B_t = \frac{S_1+S_2}{5}. \end{aligned}$$

This is the direct equivalent to the standard formula for annual rate of genetic gain by Rendel and Robertson [27], which is frequently used in livestock breeding. It equates the annual rate of genetic progress to the sum of the maternal and paternal genetic selection differentials divided by the sum of the maternal and paternal generation intervals.

Next, we turn to the calculation of $D_t$. Subtracting Eq. 4 from Eq. 5 and grouping terms yields:

15 $$\begin{aligned} D_t \,=\, &\frac{1-q}{2} D_{t-2}+\frac{(1-p)(1-q)}{2} D_{t-4}\nonumber \\&+\frac{1}{2} \Delta B_{t-3}+\frac{1-p}{2} S_1+\frac{S_2}{2}. \end{aligned}$$

Inserting Eq. 14 leads to:

16 $$\begin{aligned} D_t \,=\, &\frac{1-q}{2} D_{t-2}+\frac{(1-p)(1-q)}{2} D_{t-4}\nonumber \\&+\frac{6-5p}{10} S_1+\frac{3}{5} S_2, \end{aligned}$$

which can be solved to:

17 $$\begin{aligned} D_t=\frac{6}{p+2q-pq} \cdot \frac{S_1+S_2}{5}-\frac{p}{p+2q-pq} S_1. \end{aligned}$$

The genetic lag with controlled mating in the breeding population is thus at least $\frac{1}{10} S_1+\frac{3}{5} S_2$ if $p=q=1$ and grows larger when there is little genetic exchange between the sub-populations. As in the case of uncontrolled mating, the genetic lag $D_t$ between the sub-populations becomes eventually constant and the passive population then has the same rate of progress as the breeding population:

18 $$\begin{aligned} \Delta P_t = \frac{S_1+S_2}{5}. \end{aligned}$$

Again, we calculate the time lag *T* between the sub-populations as:

19 $$\begin{aligned} T=\frac{D_t}{\Delta B_t}=\frac{6}{p+2q-pq}-\frac{5p}{p+2q-pq} \cdot \frac{S_1}{S_1+S_2}. \end{aligned}$$

#### Summarized results

Table 2 gives an overview over the expected annual genetic gains in the breeding and the passive populations, as well as the long-term genetic and time lags between the sub-populations.

**Table 2:** Annual genetic gain in the breeding population ($\Delta B_t$) and passive population ($\Delta P_t$) as well as genetic lag ($D_t$) and time lag (*T*) between sub-populations in honeybees

	Uncontrolled mating	Controlled mating
$\Delta B_t$	$\frac{p+q}{3+3q}\cdot S_1$	$\frac{S_1+S_2}{5}$
$\Delta P_t$	$\frac{p+q}{3+3q}\cdot S_1$	$\frac{S_1+S_2}{5}$
$D_t$	$\frac{S_1}{1+q}$	$\frac{6}{p+2q-pq}\cdot \frac{S_1+S_2}{5}-\frac{p}{p+2q-pq}\cdot S_1$
*T*	$\frac{3}{p+q}$	$\frac{6}{p+2q-pq}-\frac{5p}{p+2q-pq}\cdot \frac{S_1}{S_1+S_2}$

Results are given depending on the probability *p* of a sire drone in an uncontrolled mating to come from the breeding population, the probability *q* of a queen from the passive population to have a dam from the breeding population, and maternal and paternal genetic selection differentials for reproducing colonies, $S_1$ and $S_2$

**Table 3:** Genetic selection differentials in the simulations

		$${\bar{S}}_1$$		
		Direct	Maternal	Total
Uncontr.	$r_{md}=-0.53$	0.199 (0.005)	0.278 (0.005)	0.477 (0.006)
	$r_{md}=-0.88$	− 0.058 (0.005)	0.170 (0.004)	0.112 (0.003)
Contr.	$r_{md}=-0.53$	0.397 (0.015)	0.149 (0.011)	0.546 (0.011)
	$r_{md}=-0.88$	0.351 (0.020)	− 0.123 (0.015)	0.228 (0.009)

		$${\bar{S}}_2$$		
		Direct	Maternal	Total
Contr.	$r_{md}=-0.53$	0.712 (0.033)	0.309 (0.026)	1.021 (0.026)
	$r_{md}=-0.88$	0.549 (0.037)	− 0.140 (0.028)	0.409 (0.018)

Average genetic selection differentials, ${\bar{S}}_1$ and $\bar{S}_2$, in the simulations. Averages were taken over all population sizes, numbers of isolated mating stations, values of *q* and years from 8 to 17. In brackets are the average standard deviations over the years 8 to 17

### Validation by simulation

#### Scope of the simulation

We re-investigated the simulation results of [14] and compared them with the theoretical relations derived in the previous section. The simulations had been carried out with the program BeeSim [28]. They featured breeding populations of $N_b = 500$, $N_b = 1000$, or $N_b = 2000$ colonies and passive populations of $N_p = 500$, $N_p = 1000$, or $N_p = 2000$ colonies per year. Although [14] also comprises simulations for $N_p=0$ and $N_p=\infty$, in these cases no passive queens were modeled explicitly, and thus we found these scenarios to be unfit for our analysis. The breeding population was selected for a single trait with truncation selection based on a honeybee-specific best linear unbiased prediction (BLUP) procedure [25, 29]. The selection trait had a direct (worker) and a maternal (queen) component, and two different heritabilities were modelled. In the first set-up, the maternal heritability was $h^2_m=0.53$, the direct heritability was $h^2_d=0.34$, and the correlation between the effects was $r_{md}=-0.53$. The corresponding values in the second set-up were $h^2_m=0.72$, $h^2_d=0.46$, and $r_{md}=-0.88$. Heritabilities were calculated for the selection scheme with controlled mating as described in [26, 30]. Either controlled mating took place on 5, 10, or 20 isolated mating stations, each consisting of a sister group of eight drone producing queens (DPQ), or uncontrolled mating took place. In the case of uncontrolled mating, the probability of an involved drone to have a dam from the breeding population was $p=\frac{N_b}{N_b+N_p}$. The relative proportion of passive queens with dams from the breeding population was *q*. The simulations covered the values of $q=0$, $q=0.25$, $q=0.5$, $q=0.75$, and $q=1$. The generation interval between a breeding queen and her dam was always two years, while the generation interval between a passive queen and her dam varied between one and three years (average two years), as did the average generation interval between drones in uncontrolled matings and their dams. Controlled mating was modelled with a three year age difference between the DPQ and their dam. No age difference between DPQ and their drones was assumed (Fig. 3). Thus, the population structure assumptions imposed in the theoretical derivations were largely met in the simulations, except that the simulated age structure was slightly less rigid. The simulations covered a 20-year period. See [14] for a more detailed description of the simulations.

#### Simulation outputs

For each simulated year *t*, we calculated the mean true breeding value ${\bar{B}}_t$ of the breeding colonies of that year. The corresponding values for the passive population could not be retrieved directly, because no worker groups were simulated for the passive queens [14, page 4]. Thus, we reconstructed the average passive colonies’ breeding values ${\bar{P}}_t$ from the available average breeding and passive queens’ breeding values $\bar{B}_t^Q$ and ${\bar{P}}_t^Q$ as:

20 $$\begin{aligned} {\bar{P}}_t = \frac{1}{2}{\bar{P}}_t^Q+\frac{1}{2}\left( \frac{p}{3}\sum _{i=1}^3\bar{B}_{t-i}^Q+\frac{1-p}{3}\sum _{i=1}^3{\bar{P}}_{t-i}^Q\right) , \end{aligned}$$

which reflects that a colony’s breeding value is equal to the average breeding values of the queen and of the drones that the queen mated with. Values for ${\bar{B}}_t$ and ${\bar{P}}_t$ were determined for the direct, maternal, and total breeding values, where the total breeding value of a colony was equal to the sum of its direct and maternal breeding values. The realized annual genetic progress rates and the realized genetic lag between the breeding population and the passive population were then calculated as:

$$\begin{aligned} \Delta {\bar{B}}_t&:={\bar{B}}_{t}-{\bar{B}}_{t-1},\\ \Delta {\bar{P}}_t&:={\bar{P}}_{t}-{\bar{P}}_{t-1},\\ {\bar{D}}_t&:={\bar{B}}_t-{\bar{P}}_t. \end{aligned}$$

In order to examine how well the observed values correspond to the results given in Table 2, we needed to access values for $S_1$ and $S_2$ from the simulations. Therefore, for each year $t\le 17$, we denoted by ${\bar{B}}_{1,t}$ the mean breeding value of those colonies of year *t* that were selected to produce breeding queens in year $t+2$ and by ${\bar{B}}_{2,t}$ the mean breeding value of those colonies that were selected to produce drone producing queens in year $t+3$. Then, for $j=1,2$:

$$\begin{aligned} {\bar{S}}_{j,t}:={\bar{B}}_{j,t}-{\bar{B}}_t. \end{aligned}$$

With these resulting values for $\Delta {\bar{B}}_t$, $\Delta {\bar{P}}_t$, ${\bar{D}}_t$, ${\bar{S}}_{1,t}$, and ${\bar{S}}_{2,t}$, we investigated, how well the simulated data represented the relations of Table 2.

## Results

### Values of ${\bar{S}}_1$ and ${\bar{S}}_2$

The attained values of ${\bar{S}}_{1,t}$ and ${\bar{S}}_{2,t}$ depended mainly on the correlation between direct and maternal effects and, in the case of ${\bar{S}}_{1,t}$, on whether controlled or uncontrolled mating took place (see Table 3). The different values of $N_b$, $N_p$, and *q* had only negligible effects on ${\bar{S}}_{1,t}$, and also the effects on ${\bar{S}}_{2,t}$ were small. Regarding the birth year *t*, we observed that after some variability in the initial years, the values of ${\bar{S}}_{1,t}$ and ${\bar{S}}_{2,t}$ appeared to no longer depend on *t* for $t\ge 8$, which justifies the assumption of the genetic selection differentials being constant over time.

For the trait with a moderate correlation between maternal and direct effects, $r_{md}=-0.53$, the genetic selection differential for total breeding values for dam replacement, ${\bar{S}}_1$, was 14% higher when mating was controlled than under free mating conditions. For the trait with a strong negative correlation between effects, $r_{md}=-0.88$, the genetic selection differential ${\bar{S}}_1$ even doubled with controlled mating. The genetic selection differentials for the production of drone producing colonies, ${\bar{S}}_2$, were generally higher than those for dam replacement, reflecting the higher selection intensities for this purpose. This was more pronounced for the trait with a moderate correlation between maternal and direct effects, $r_{md}=-0.53$, for which ${\bar{S}}_2$ was 87% higher than ${\bar{S}}_1$, than for the the trait with $r_{md}=-0.88$, for which the corresponding number was 79%. In the case of a strong negative correlation between maternal and direct effects, $r_{md}=-0.88$, negative genetic selection differentials were observed for either the maternal or the direct effects. As already observed in [14], selection with uncontrolled mating worked against the direct effect, while with controlled mating, genetic progress in the maternal effect was sacrificed in favor of the direct effect.

### Uncontrolled mating

Exploiting the observation that ${\bar{S}}_{1,t}$ and ${\bar{S}}_{2,t}$ were largely independent from *t* from year 8 on, we defined $\bar{S}_1$ and ${\bar{S}}_2$ as the averages of the respective genetic selection differentials of years 8 to 17.

**Fig. 4:**
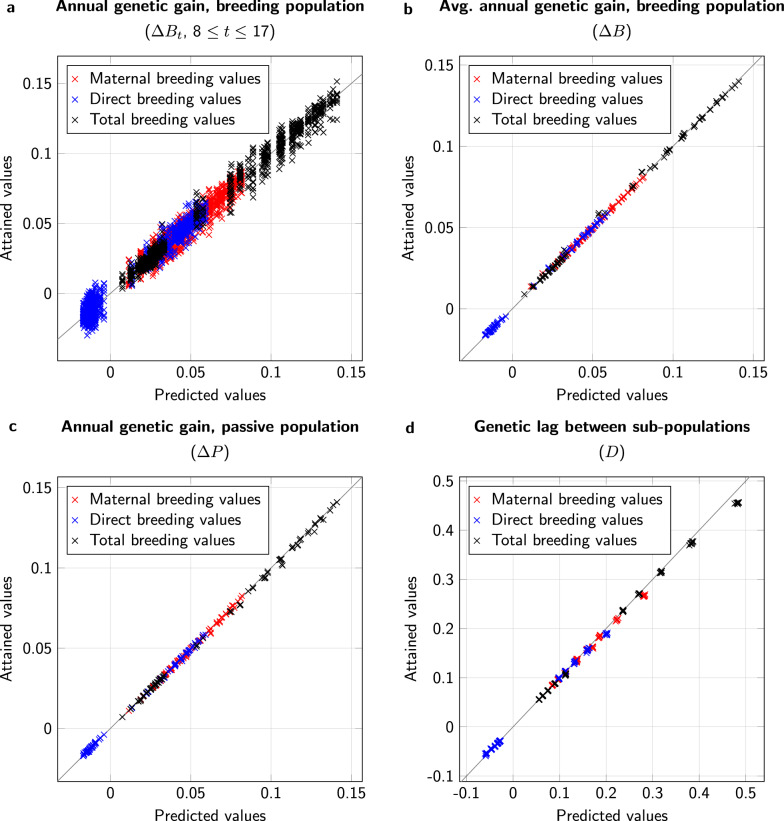
Comparison of predicted (horizontal axis) and simulated values (vertical axis) for genetic progress. a: Annual genetic improvement in the breeding population, $\Delta B_t$, in individual years 8 to 17. b: Average annual genetic improvement in the breeding population, $\Delta B$, over the years 8 to 17. c: Average annual genetic improvement in the passive population, $\Delta P$, over the years 8 to 17. d: Average genetic lag, *D*, over the years 8 to 17. Values are highlighted for maternal, direct, and total breeding values; diagonal equality lines are drawn for orientation

When we compared the attained values of $\Delta {\bar{B}}_t$ ($8\le t\le 17$) with the predictions $\frac{p+q}{3+3q}{\bar{S}}_1$ for all different settings and all types of breeding values (maternal, direct, total), we found a regression coefficient of 0.994 with a coefficient of determination (squared correlation) of 0.980 (Fig. 4a). When we compared the prediction with the average observed values $\Delta {\bar{B}}=\frac{1}{10}\sum _{t=8}^{17}\Delta \bar{B}_t$, the coefficient of determination improved to 0.999 (Fig. 4b).

In addition, for the annual improvement of the passive population and the genetic lag between the sub-populations, we found that the predictions matched the observations from the simulations (Fig. 4c and d). Comparing $\Delta \bar{P}=\frac{1}{10}\sum _{t=8}^{17}\Delta {\bar{P}}_t$ with the predicted $\frac{p+q}{3+3q}{\bar{S}}_1$, we found a regression coefficient of 0.990 and a coefficient of determination of 0.999. The corresponding values for ${\bar{D}}=\frac{1}{10}\sum _{t=8}^{17}{\bar{D}}_t$ were 0.964 for the regression coefficient and 0.999 for the coefficient of determination.

### Controlled mating

**Fig. 5:**
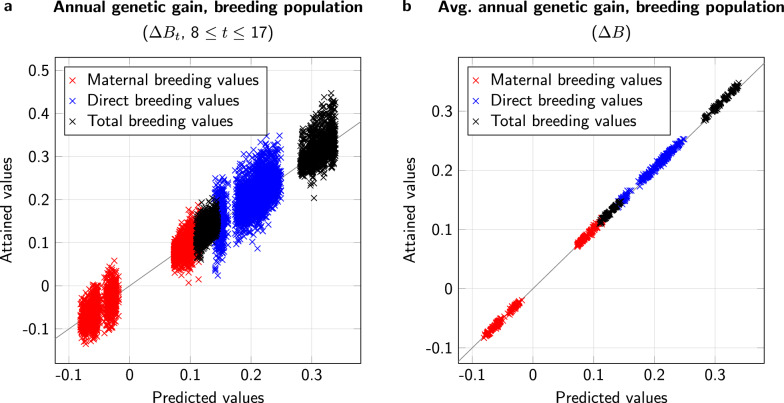
Comparison of predicted (horizontal axis) and simulated values (vertical axis) for genetic progress. a: Annual genetic improvement in the breeding population, $\Delta B_t$, in individual years 8 to 17. b: Average annual genetic improvement in the breeding population, $\Delta B$, over the years 8 to 17. Values are highlighted for maternal, direct, and total breeding values; diagonal equality lines are drawn for orientation

With controlled mating, the results for the breeding population were similar to those obtained for uncontrolled mating. Comparing the values of $\Delta {\bar{B}}_t$ ($8\le t\le 17$) with the predictions $\frac{ {\bar{S}}_1+{\bar{S}}_2}{5}$, we found a regression coefficient of 1.016, with a coefficient of determination of 0.948 (Fig. 5a). When we compared the prediction with the average observed values $\Delta {\bar{B}}$, the coefficient of determination became practically 1 (deviation $<10^{-3}$) (Fig. 5b).

**Fig. 6:**
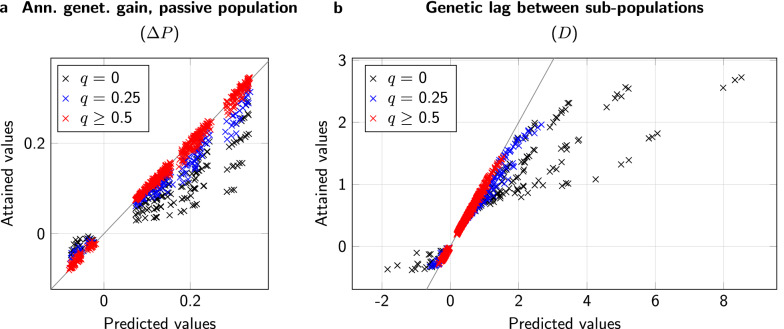
Comparison of predicted (horizontal axis) and simulated values (vertical axis) for genetic progress. a: Average annual genetic improvement in the passive population, $\Delta P$, over the years 8 to 17. b: Average genetic lag, *D*, over the years 8 to 17. Values are highlighted for maternal, direct, and total breeding values; diagonal equality lines are drawn for orientation

With regard to the passive population, the accordance of predicted genetic gain with realized genetic gain depended mainly on the proportion *q* of passive queens reared from breeding colonies (Fig. 6a). For $q=0$, in comparison with the averaged values $\Delta {\bar{P}}$, the prediction, $\frac{ {\bar{S}}_1+\bar{S}_2}{5}$, severely overestimated the actual genetic gain, with a regression coefficient of 0.617, and had a coefficient of determination of only 0.841. For $q=0.25$, the regression coefficient increased to 0.827 and the coefficient of determination improved to 0.975, and finally for $q\ge 0.5$, we found a regression coefficient of 0.984, with a very good coefficient of determination of 0.996.

When we considered the genetic lag ${\bar{D}}$, we found a situation that was similar to our observations regarding $\Delta {\bar{P}}$ (Fig. 6b). For $q=0$, we found a severe underestimation of ${\bar{D}}$, with a regression coefficient of 0.369 and a relatively low coefficient of determination of 0.881. For $q=0.25$, these values improved to 0.782 and 0.987, respectively. Finally, for $q\ge 0.5$, we arrived at a regression coefficient of 0.960 with a coefficient of determination of 0.998.

## Discussion

### Consistency between theory and simulations

#### Uncontrolled mating

When mating was uncontrolled, the model that we derived matched the observations from the simulations very well. The method that we used to derive our theoretical predictions shows great similarities to the gene-flow method [23, 24], which is a well-established model in animal breeding. However, unlike in Hill’s original work [23], the method was used to assess the influences of different sub-populations, rather than the influences of different age-cohorts.

In our theoretical derivations, we assumed that, with uncontrolled mating conditions, drones always came from two-year old dam queens, whereas in the simulations, the generation interval between drones and their dams could vary between one and three years. In order to model this situation more accurately, one would have to replace $B_{t-4}$ and $P_{t-4}$ in Eqs. 1 to 4 by $\frac{B_{t-3}+B_{t-4}+B_{t-5}}{3}$ and $\frac{P_{t-3}+P_{t-4}+P_{t-5}}{3}$, respectively. However in our model, we assumed that genetic gain will be linear after some years, so that $\frac{B_{t-3}+B_{t-4}+B_{t-5}}{3}=B_{t-4}$ and $\frac{P_{t-3}+P_{t-4}+P_{t-5}}{3}=P_{t-4}$. Similar considerations apply regarding the variable age structures of passive queens as dams of passive queens.

The clearly defined generation intervals of two years simplified the terms in our derivations. However, we see no theoretical restriction that would prevent our method of derivation to be applied to other age structures, including overlapping generations.

#### Controlled mating

The high prediction accuracy that was observed for the development of the breeding population with controlled mating was expected. As pointed out in the Methods section, the corresponding formula Eq. 14 is a direct analogue of the famous equation of Rendel and Robertson [27], which is a standard tool in animal breeding.

For small values of *q*, i.e. when passive populations were mainly self-sufficient regarding dam queen production, the predictions overestimated the realized genetic progress in the passive population. Thus, the predictions for the genetic lag $D_t$ between the breeding population and the passive population were also biased. However, Plate *et al.* [14] already suggested that for small values of *q*, the passive population will simply take longer than the simulated 20 years to reach the rate of genetic gain of the breeding population. Indeed, it can be shown that for smaller *q*, the relevant recursion Eq. 16 has a lower convergence rate. The finding that, for small values of *q*, the annual genetic gain in the passive population still increased after year 8 can fully explain the overestimation of both genetic progress in the passive population and genetic lag between the breeding population and the passive population. We expect that, with a longer time-frame, we would find good matches between predicted and observed values also for small *q*.

Prediction of the genetic lag $D_t$ with controlled mating (Eq. 17) was considerably more complex than its counterpart with uncontrolled mating (Eq. 7). This reflects the great differences between the paternal paths of inheritance with controlled and uncontrolled mating.

In general, one can state that in breeding schemes with controlled mating and sufficiently high values for *q*, the derived formulas gave a good description of the genetic development of the breeding and passive population. However, they do not account for inbreeding or genetic drift and therefore will always predict a linear genetic gain over time. Long-term simulation studies in honeybees and other species have shown that such a behavior over many generations is unrealistic [14, 28, 31–33].

### Applications of the model

#### Assessment of input variables

Our model predicts the genetic progress of honeybee populations based on the parameters *p*, *q*, $S_1$, and $S_2$. Thus, when our model is used by breeders to plan new selection schemes, it will be necessary to obtain realistic values for these input variables. Values for *q* are a direct consequence of the breeding plan. Values for *p* may be estimated from the relative numbers of breeding and passive colonies in the breeding area. However, one should note that in particular for *Varroa* tolerance breeding, there may be a reproductional advantage for drones from colonies with higher breeding values, which may increase the probability *p* for drones from breeding colonies to mate successfully [34]. Furthermore, the assumption that with uncontrolled mating, breeding and passive queens have the same probability *p* to mate with a drone from a breeding colony could be questioned if breeding and passive colonies are not evenly distributed over the breeding area.

Reliable values for $S_1$ and $S_2$ may be slightly harder to obtain. After a breeding program has run for a couple of generations, these values may be retrieved from the estimated breeding values, similar to how we retrieved them from our simulations. Before the beginning of the actual breeding, $S_1$ and $S_2$ may be derived from the genetic parameters and the intended selection intensity. If the true breeding values of colonies are normally distributed with variance $\sigma ^2$ and breeding values are estimated with accuracy $\rho$, then truncation selection with intensity *i* will lead to a genetic selection differential of [35]:

21 $$\begin{aligned} S=i\cdot \rho \cdot \sigma . \end{aligned}$$

However, it should be noted that the $\sigma$ in Eq. 21 will in general differ from the additive genetic standard deviation $\sigma _A$, because of the different variance structures for queens and worker groups [26] and because for a population under selection, $\sigma$ is reduced by the Bulmer effect [16]. Eq. 21 can be used to explain the observation that $S_1$ had larger values with controlled mating (Table 3). Controlled mating leads to a more accurate estimation of breeding values (larger $\rho$); in addition, the variance of the colonies’ true breeding values, $\sigma$, is enhanced with controlled mating due to the positive correlations between the true breeding values of drones on a mating station.

#### Generalizations

To our knowledge, there has not been any previous analytical prediction of genetic gain in passive populations of honeybees. However, in analytical investigations of nucleus breeding schemes in cattle, similar approaches based on systems of recurrence equations, also proved parallel genetic progress in the nucleus and the base populations [21, 22]. Analogous recurrence equations of similar structure can be formed in many other situations, allowing far-reaching generalizations of the presented results. For example, one can expect that whenever there is some (ever so small) genetic exchange between two or more animal populations, they will eventually show parallel genetic development, i.e. they can diverge from one another only up to a certain limit.

It is conceivable to derive similar recursion formulas to quantify the genetic improvement that can be achieved by the introduction of controlled mating in other agricultural species. In particular, such results may have implications for breeding activities in various countries, for which previous studies have shown that a lack of controlled mating undermines efforts in livestock breeding [36–38].

In the more concrete setting of honeybee breeding, the presented model allows an extrapolation of the simulation results in [14] to the diverse cases of actual breeding populations, each with its own set of parameters, such as population size or genetic exchange rates between the breeding and passive populations. This is especially important since the sizes of the passive populations relative to the breeding populations as they were used in the simulation studies in [14] were suitable to illustrate general phenomena but not necessarily realistic. For example, the large Central European breeding population of *Apis mellifera carnica* managed by beebreed.eu comprises about 8000 breeding queens per year [39], but the German Beekeepers’ Association (DIB) estimates the total number of honeybee colonies in Germany to be approximately 1,000,000 [40]. Applying our formulas to specific real cases allows, for example, the expected benefits from the introduction of controlled mating in breeding systems that have previously relied on free-mated queens to be quantified.

It is likely that the derivations of the formulas presented here can be adapted to perform other investigations on honeybee breeding. A further possible generalization would be an investigation of cases where only some of the breeding queens undergo controlled mating or where some of the mating stations are not entirely secure.

Another application might arise from the adaptation of nucleus breeding systems for honeybees. For example, testing honeybees for the trait suppressed mite reproduction (SMR), indicating the colony’s ability to cope with the parasite *Varroa destructor*, involves a complex procedure that is likely to be practiced only by few breeders [41]. However, the genetic material of colonies who excel in SMR is spread into the general breeding or passive population [42]. This is factually the structure of a nucleus breeding program and the dependencies between the nucleus population bred for SMR and the broader breeding population can be expressed in formulas similar to those presented here.

## Conclusions

Our model explained well the genetic dynamics for honey bee populations where the breeding sub-population is under uncontrolled mating. With the limitation of a long convergence time in case of little queen transfer from the breeding to the passive population, this also holds true for breeding populations with controlled mating. The model allows the mechanisms behind the observations of [14] to be better understood and generalizations to a wide range of honeybee populations are now possible.

## Data Availability

The datasets used and/or analysed during the current study are available from the corresponding author on reasonable request. The source code of the simulation program BeeSim is available at https://doi.org/10.5061/dryad.1nh544n.
