# Correction to: A theoretical derivation of response to selection with and without controlled mating in honeybees

**DOI:** 10.1186/s12711-021-00617-2

**Published:** 2021-02-24

**Authors:** Manuel Du, Richard Bernstein, Andreas Hoppe, Kaspar Bienefeld

**Affiliations:** Institute for Bee Research Hohen Neuendorf, Friedrich-Engels-Str. 32, 16540 Hohen Neuendorf, Germany

## Correction to: Genet Sel Evol (2021) 53:17 https://doi.org/10.1186/s12711-021-00606-5

Following publication of the original article [1], the authors reported an error in Table 1. The lines are not correctly aligned.

The correct Table 1 is given below:

**Table 1:** Variable definitions

Variable	Definition
*t*	Subscript referring to the year. If variables are used without this subscript it is assumed that they are constant for all years
$B_t$ / $P_t$	Average true breeding value of breeding/passive colonies of year *t*
$\Delta B_t$ / $\Delta P_t$	$=B_t-B_{t-1}$ (resp. $=P_t-P_{t-1}$) Annual genetic gain among the breeding/passive colonies
$D_t$	$=B_t-P_t$ Genetic lag between the breeding population and the passive population
*T*	Time lag, time it takes for the passive population to reach the genetic level of the breeding population
$p_t$, *p*	Probability that a sire drone in an uncontrolled mating comes from a breeding colony
$q_t$, *q*	Proportion of passive queens with a dam queen from the breeding population
$S_{1,t}$, $S_1$	Genetic selection differential of colonies selected for breeding queen production
$S_{2,t}$, $S_2$	Genetic selection differential of colonies selected for DPQ production
$N_b$ / $N_p$	Number of breeding/passive colonies per year
$h^2_m$ / $h^2_d$	Maternal/direct heritability
$r_{md}$	Correlation between maternal and direct effects
